# Design and effects of the teacher-student interaction model in the online learning spaces

**DOI:** 10.1007/s12528-022-09348-9

**Published:** 2022-12-31

**Authors:** Youru Xie, Yuling Huang, Wenjing Luo, Yucheng Bai, Yi Qiu, Ziru Ouyang

**Affiliations:** 1 School of Educational Information Technology, South China Normal University, Guangzhou, China; 2 ZhuJiang Hospital of Southern Medical University, Guangzhou, China; 3 NanCheng No.1 Junior Middle School, Dongguan, China; 4 University of Birmingham, Birmingham, UK

**Keywords:** Teacher-student interaction, Online learning spaces, Model construction, Learning effect

## Abstract

The interaction between teachers and students is vital for promoting teaching quality. Online learning spaces have various features that can support teacher-student interaction in online learning contexts. In this study, a preliminary model was developed by analyzing the principles underlying the interaction between teachers and students and the support features of online learning spaces. Then, the interaction model was refined and validated in three rounds of teaching practice involving 31 college students. A real-time dynamic artificial intelligence analysis system was used to analyze the teacher-student interaction during three rounds of design-based research. The results showed that the model significantly fostered students’ engagement during the interaction. Moreover, students significantly improved their final exam scores and their innovative problem-solving ability after the intervention.

## Introduction

Today, higher education is under great pressure and faces considerable challenges. Teachers must not only help students acquire existing knowledge and skills, but also and more importantly, they must enable students to apply and generate new ideas. Fostering an effective teacher-student interaction can positively impact learning in the higher education setting (Zhang, 2019). The teacher-student interaction refers to a process in which teachers and students use various learning resources to fulfill their specific roles and responsibilities through different teaching and learning activities. These activities use various behaviors in the process of learning to achieve teaching goals through mutually influencing interaction. However, this kind of interaction does not appear often in the current classroom setting in colleges, and problems persist, e.g., the attitude of students towards learning is inactive or the degree of interaction is unclear. Therefore, it is important to reconsider the principle of interaction between teachers and students in today's information era (Dobrotina et al., 2016).

The use of information technologies may help to foster active teacher-student interactions more effectively (Zhang, 2019). Explore the application of online learning spaces is of great significance for supporting the teacher-student interaction. Services such as resource sharing, teaching support, analysis and evaluation, as well as teaching management provided by the online learning space greatly support the smooth implementation of teacher-student interaction activities. The resource-sharing service may help students to retrieve resources and push resources in a personalized manner, thus achieving the goal of co-creating and sharing. The teaching support service can support teachers manage the teaching process, help students to grow their knowledge, and provide intelligent tutoring services that can also reduce teachers' pressures to a certain extent. The analysis and evaluation service provides teachers with intelligent diagnoses and analyses about learning outcomes and provides students with immediate learning alerts. These resources may help both teachers and students to rapidly adjust their teaching and learning methods. Accurate student learning data, together with intelligent performance management systems, may help teachers to better manage the classroom and provide a more harmonious learning atmosphere for students.

To explore how to better support teacher-student interaction and effectively gain knowledge in online learning spaces, an in-depth theoretical and practical research on the teacher-student interaction model was conducted. A set of theoretical frameworks for the teacher-student interaction was established, and the principles of teacher-student interactions and the functional support of online learning spaces for teacher-student interaction were clarified. Then, the teacher-student interaction model in online learning spaces was constructed, and the interaction mechanism, interactive behaviors, tools support, and implementation strategies of the model were further improved through a design-based study.

The effectiveness of the teacher-student interaction model was examined from three aspects based on online learning spaces: the teacher-student interaction, students' ability to solve innovative problems, and learning performances. Firstly, the S-T curve of teacher-student interaction shows that the degree of teacher-student interaction increased. Secondly, the independent sample T test results of the innovation problem solving ability scale identified a significant difference between pre-test and post-test periods. Thirdly, the results of the‾X-*S* plane analysis model show that the average level of the final examination scores of the subjects was high.

Therefore, the teacher-student interaction model in the online learning spaces can effectively enhance the teacher-student interaction, and help students to improve their ability to solve problems innovatively, achieve higher cognitive attainment, and improve their learning performance.

## Literature review

### Teacher-student interaction

Teachers and students develop a bilateral and interactive relationship in the education process via interaction and mutual influence (Ye & Pang, 2001). Dong et al. (2020) confirmed that prior knowledge, cognitive load, and help-seeking strategies affect student learning engagement in teacher-student interactions. Various strategies can be used to improve the teacher-student interaction at various teaching stages, examples of which are strategies for enhancing collaboration and interaction awareness, presentation and analysis strategies, practical application strategies, self-assessment and reflection strategies, and continuous contact strategies (Dao, 2020).

Moreover, researchers have developed teacher-student interaction models to facilitate interactions in a more structured manner. For example, Song et al. (2020) developed a teacher-student interaction measurement model for online teaching from the perspectives of pedagogical existence and social existence. Sybing (2021) constructed a conversational model for teacher-student interactions that can determine dialogue opportunities arising during in-class interactions and the relevant characteristics of active dialogue development.

In summary, although numerous studies on teacher-student interactions have been conducted, further studies are needed to explore how to analyze and foster more effective teacher-student interactions in online environments.

### Online learning spaces

Research on online learning spaces mainly focuses on four aspects: theoretical research, design and development, analysis of learning behavior, and teaching practices. In their theoretical research, Jin et al. (2021) discussed the situation, key issues, and innovative applications of early academic warnings and interventions in online learning spaces. Costley (2020) analyzed the relationship between online learning cognitive load and Delman's cognitive load, and explored how to use cognitive learning strategies to alleviate this relationship. In terms of design and development, Yang et al., (2021a, 2021b) explored the construction and application of online learning spaces during the COVID-19 pandemic and the post-pandemic era. In terms of learning behavior analysis, Thai et al. (2020) compared four learning environments regarding students' learning performance. Their study identified a significant positive impact on students' performance when learning in blended learning and flipped classroom environments. In terms of teaching practices, Taghizade et al. (2020) examined the impact of using a teaching model on learners' perception status and advanced learning outcomes in online learning spaces.

In summary, as an integrated virtual space that supports online and offline as well as synchronous and asynchronous teaching, the online learning space provides important support for improving the quality of teacher-student interactions. However, there is a lack of research studies that examine the inherent functional supports of online learning spaces from the perspective of teacher-student interaction. The present study aims to fill these knowledge gaps.

## Model construction

### Principles and analysis of teacher-student interaction

#### Elements of teacher-student interaction and their relationships

**Fig. 1:**
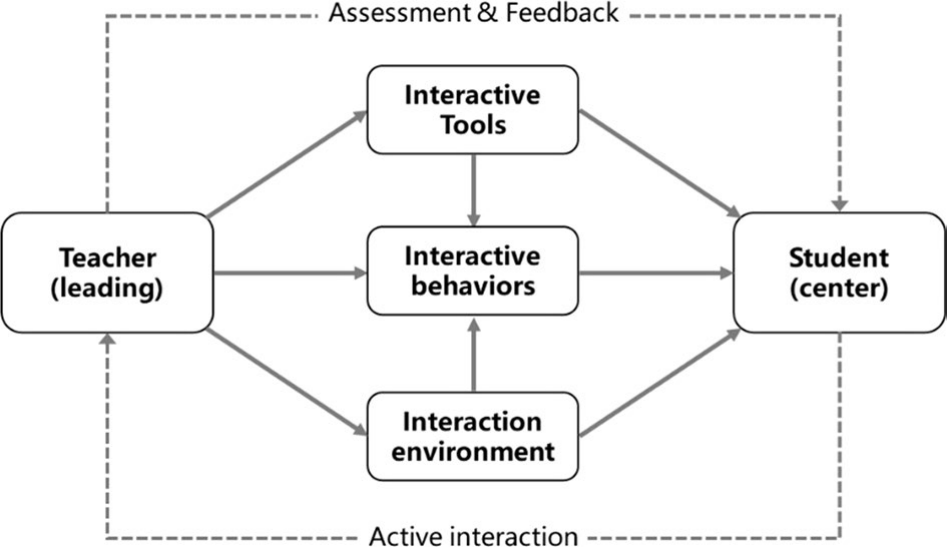
Elements of the teacher-student interaction and their relationship

An analysis of the literature identified the following four elements of teacher-student interaction: subject, behavior, tools, and environment. The elements of teacher-student interaction and their relationships are depicted in Fig. 1. At the interactive subject, students are at the central position and teachers dominate teaching. Both teachers and students use various tools to carry out activities in the corresponding environment, thus showing different teaching behaviors.

*Subjects* Teachers and students are the two fundamental elements of teaching activities.

*Behaviors* Teachers and students can freely communicate, discuss, and collaborate to share views and thoughts. Specifically, teachers’ behaviors include questioning, providing feedback, direct teaching, demonstrations, classroom supervision, and classroom control. Students’ behaviors include responding to teachers’ questions, active questioning, discussing, practicing, and watching demonstrations.

*Tools* Many tools are used for live, interactive, and synchronized collaboration, as well as discussion and communication, information collection, and classroom management. Live interactive tools enable teachers and students to interact online in real-time, which enhances immersion between teachers and students in online learning spaces. Synchronous collaboration tools help teachers organize students to carry out group cooperation activities while achieving smooth communication and efficient collaboration. Discussion and communication tools help both teachers and students share and disseminate knowledge. Information collection tools help teachers to quickly grasp the overall learning situation and effectively improve teaching efficiency. Classroom management tools integrate multiple functions such as online correction, data statistics, and resource management, which can help teachers to effectively manage the classroom.

*Environment* This refers to the physical environment where learning occurs, the technologies adopted, and the resources that support the teacher-student interaction. Online learning spaces are the primary pedagogical environments, and can provide various services such as resource sharing, teaching support, analysis and evaluation, as well as education management. Resource-sharing services can help students retrieve resources and personalize them to achieve the co-creation and sharing of resources. Teaching support services can support teachers manage their teaching, help students manage knowledge, and relieve teachers’ teaching pressure to a certain extent. Analysis and evaluation services provide teachers with intelligent academic diagnosis and analysis tools and provide students with real-time learning alerts, both of which help teachers and students to rapidly adjust teaching methods and learning methods. Precise attendance data and the intelligent performance management system may assist teachers in managing the classroom and provide students with a more harmonious learning atmosphere.

**Fig. 2:**
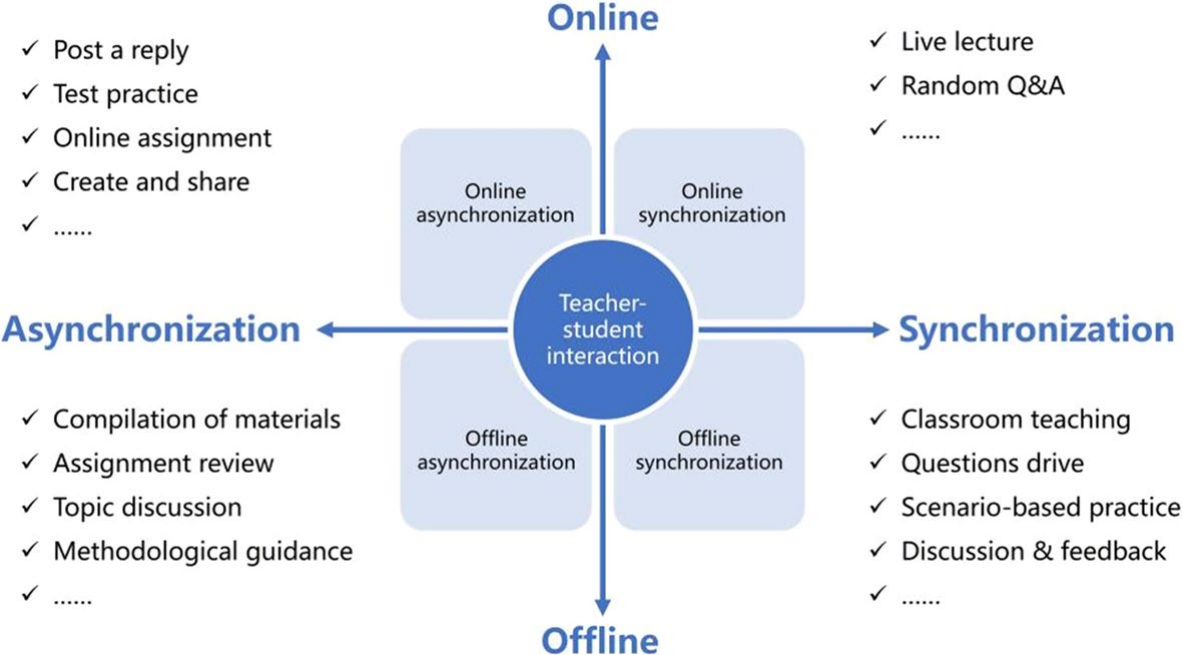
Four types of teacher-student interactions

According to the two dimensions of time and place, teacher-student interactions were divided into four categories: online synchronization, online asynchronization, offline synchronization, and offline asynchronization, as shown in Fig. 2.

### Features of online learning spaces in supporting teacher-student interaction

**Fig. 3:**
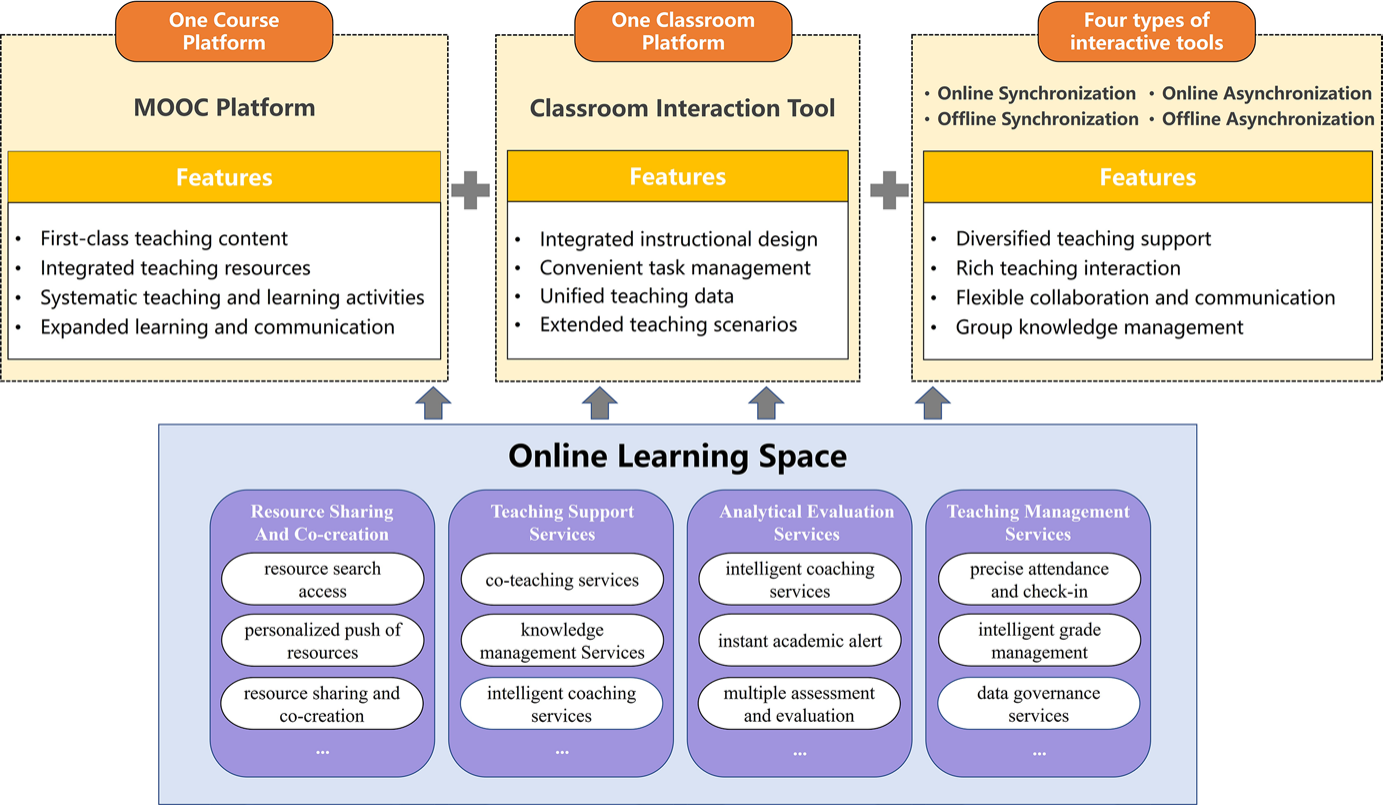
Application methods of online learning spaces to support teacher-student interactions

In this study, the following features of three teaching elements were identified: one course platform, one classroom platform, and four types of interactive tools. Application methods are proposed for online learning spaces to support the teacher-student interaction in these three elements, as shown in Fig. 3.

*One course platform* is the MOOC platform of China University, which provides students with first-class, high-quality teaching content. During online teaching, teachers can incorporate rich and diverse teaching resources such as micro-lessons, classroom videos, and hypertext. Moreover, the platform can be used to organize systematic and complete teaching activities, such as viewing resources, discussions and replies, tests, and exercises. Teachers can also use this platform to expand online multi-party learning and communication, as well as to promote equal interaction and communication among students, teachers, and other education workers.

*One classroom platform* is the MU Classroom platform, which is an online and offline blended smart teaching tool developed based on the MOOC platform of China University. During offline teaching, teachers rely on MU Classroom to carry out integrated teaching design, manage tasks, view online and offline learning data in real-time, and adjust both content and methods of teaching accordingly. Moreover, MU Classroom helps teachers synchronize offline interaction data with online course teaching data on the MOOC platform of China University, thus forming a closed loop for online and offline teaching. Furthermore, the MU Classroom platform supports teacher-student interactions through features such as in-class tests, random roll-calls, class questionnaires, and assignments, thereby creating a variety of interactive scenarios.

*Four types of interaction tools* are online synchronous interactive tools, online asynchronous interactive tools, offline synchronous interactive tools, and offline asynchronous interactive tools. The interactive tools complement the course platform and classroom platform, compensate for the functional limitations of the platform, and expand the diversity of interaction forms. These tools provide a variety of teaching and learning support, rich teaching and learning interactions, flexible collaboration, and group knowledge management. These are all important for facilitating quality and effective teacher-student interactions.

### The Preliminary teacher-student interaction model

**Fig. 4:**
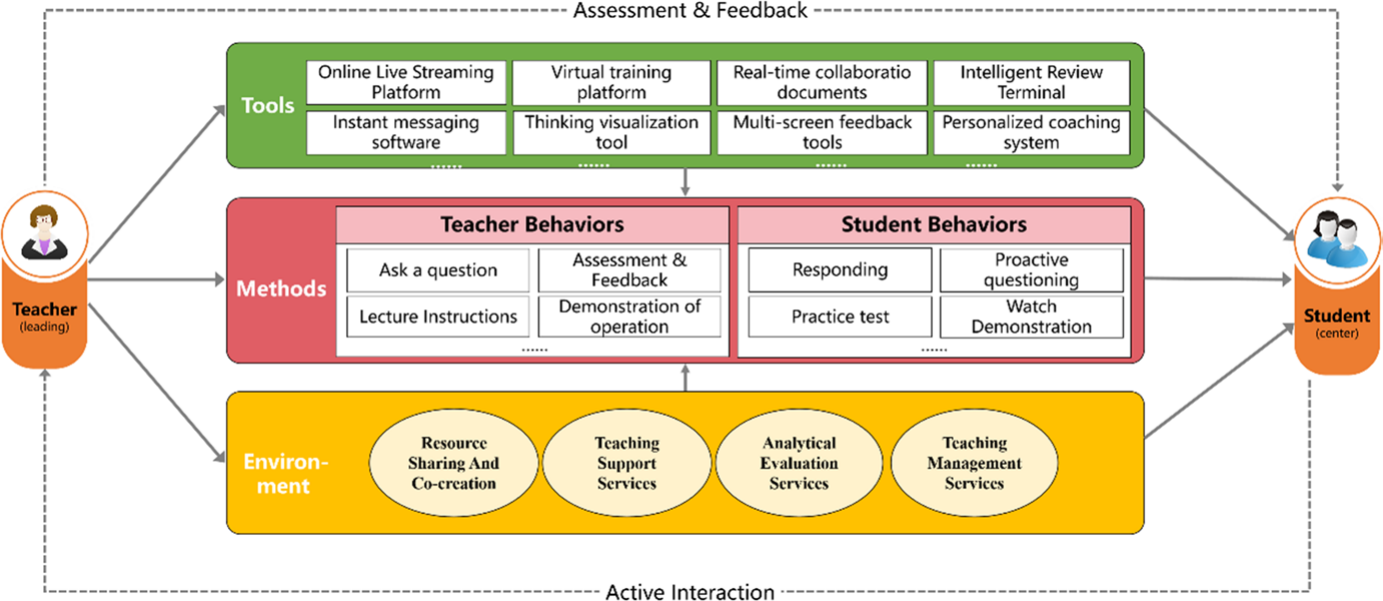
Preliminary model of teacher-student interaction in online learning spaces

Based on previous analyses, a preliminary model of the teacher-student interaction in online learning spaces is proposed and shown in Fig. 4. The model shows the relationship between the elements of teacher-student interaction and the functional supports for online learning spaces.

## Model refinement

### Research design

#### Participants

The sample for this study consisted of 31 undergraduate students enrolled in the Research Methods in Educational Technology course. The students had blended learning experiences in other prerequisite courses. The course was taught online using the Educational Technology Research Methods course on the MOOC platform of China University, and offline using MU Classroom. The course was supplemented by other interactive tools as appropriate.

#### Research methods

A design-based research method was adopted and the teacher-student interaction model in the online learning spaces was refined via three rounds of iterative modifications. The design-based research schedule is presented in Table 1.

**Table 1:** Design-based research schedule

Design-based research	Purpose of the study	Teaching content	Teacher-student interaction behaviors	Teacher-student interaction tools
First round September 10, 2020–October 15, 2020	To analyze the institution of teacher-student interaction	① Overview of educational technology research methods ② Educational technology evaluation research	Recorded lectures, posting responses, learning feedback, critique feedback, classroom workshops, data compilation	MOOC platform of China University, MU Classroom, Live interactive tools, Discussion and communication tools
Second round October 15, 2020–October 22, 2020	To refine the interactive behaviors and tools supported in the model	① Research on online teaching in the period of the COVID-19 pandemic prevention and control ② Content analysis	Recorded lectures, posting responses, learning feedback, critique feedback, classroom workshops, data compilation	MOOC platform of China University, MU Classroom, Synchronous collaboration tools, Discussion and communication tools
Third round October 29, 2020–November 19, 2020	To propose an implementation strategy of the model based on the first two rounds	① Statistical processing of research data ② Educational technology action research	Recorded lectures, posting responses, test exercises, task management, learning feedback, topic discussion, classroom workshops, face-to-face Q&A, knowledge construction, knowledge management	MOOC platform of China University, MU Classroom, Information collection tools, Classroom management tools

### Instruments

*Real-time dynamic artificial intelligence (AI) analysis system* A real-time dynamic AI analysis system for teaching and learning was used to analyze classroom interaction behaviors. This system was also used to both collect and analyze classroom discourses as well as various teaching data (such as the percentage of teacher-student speech and response rate) generated in the classroom through AI analysis and computational models. Analysis reports were also formed with the same system. System-generated S-T curves and the data of Flanders analysis were used to examine whether the teacher-student interaction has improved.

*The Scale of Creative Problem-solving Skills (CPS)* Based on the CPS model (Torrance, 1972), the scale of CPS was developed in reference to the evaluation index of problem-solving ability for collaborative knowledge construction in online classrooms (Xie, 2009) and the evaluation index of college students' innovation ability (Cao, 2008). The CPS evaluation scale was transformed into an electronic questionnaire which was sent to students through a link or QR code. One hundred valid data sets were obtained. A reliability analysis of the CPS evaluation scale was conducted. The Cronbach's α reliability coefficient was 0.968, indicating that the evaluation scale had good internal consistency and that the measurement model was credible.

*The Final Exam* The final exam focused on assessing students' comprehension ability. The questions included judgment questions, short-answer questions, and design questions, and the paper was moderately difficult with a full score of 100 points. This final examination paper had gone through the modulation process by the Academic Affairs Office of South China Normal University, indicating that the number of questions and the level of difficulty of the examination papers met the examination requirements and did not deviate from papers of previous years.

### First round in design-based research: teacher-student interaction mechanism in online learning spaces

The teaching content of the first round was the Overview of Educational Technology Research Methods and the Educational Technology Evaluation Research. The design of teacher-student interaction was based on the model of teacher-student interaction in online learning spaces, as shown in Fig. 4.

**Fig. 5:**
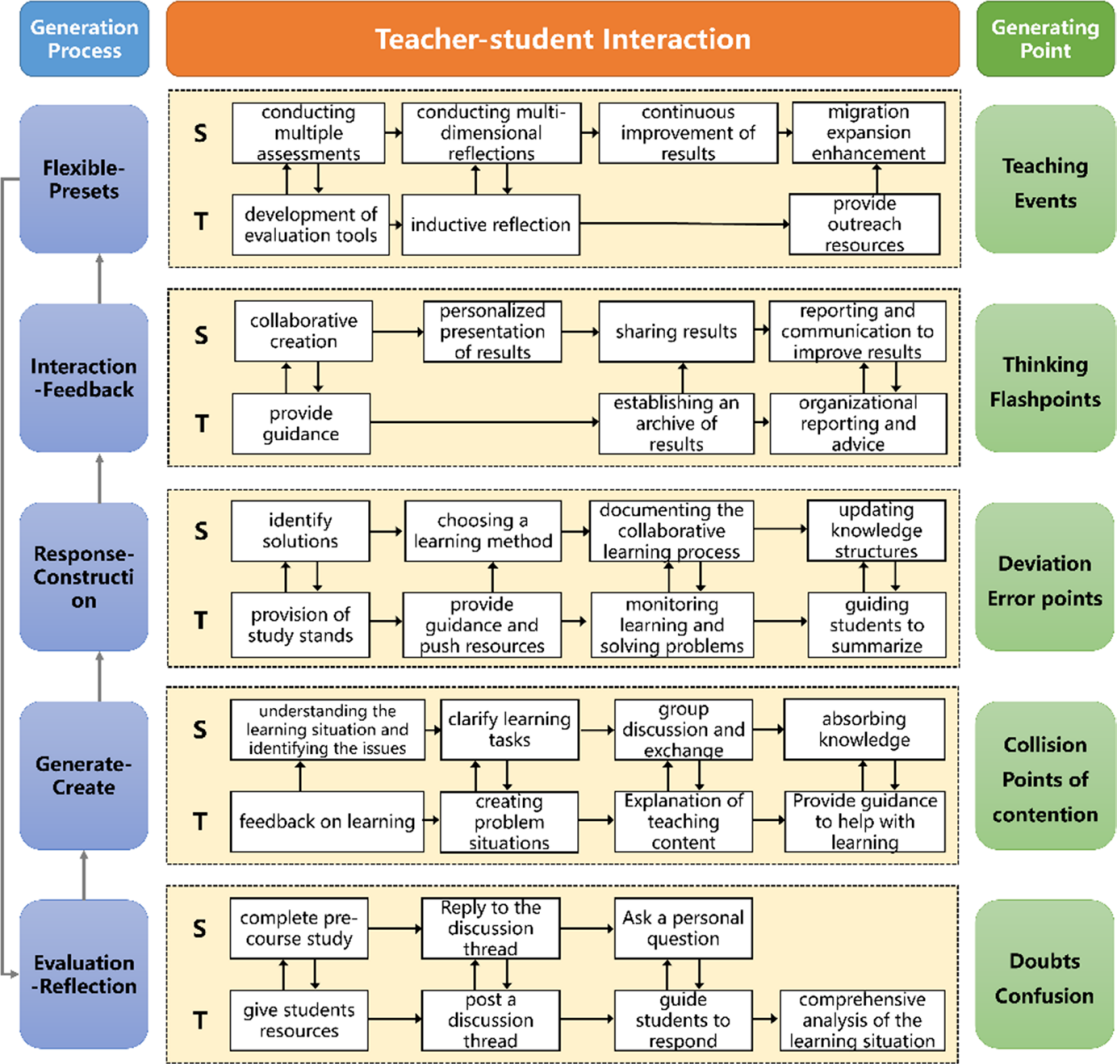
Teacher-student interaction mechanism based on online learning space

Certain scholars proposed that the teaching path should include the following five stages: Flexible-Presets, Interaction-Feedback, Response-Construction, Generate-Create, and Evaluation-Reflection (Xie et al., 2016)*.* Based on this proposal, the platform data were analyzed. The results showed that the learning effect of applying this model was promising, but the data also identified problems: ① In the Flexible-Presets stage, students were able to respond to the discussion topics posted by the teacher as requested, but most students copied the textbook content without expressing their own understanding and digestion. ② In the Interaction-Feedback stage, because of time constraints, teachers could only randomly guide individual groups in class, making it difficult for students to grasp the progress and ensure the quality of the overall discussion. ③ In the Response-Construction stage, teachers guided students to review old knowledge and learn new knowledge. However, the various levels of student understanding and the inconsistent progress of collaborative group construction make it difficult for teachers to propose individualized, targeted learning scaffolds for individual students and groups. ④ In the Generate-Create stage, teaching activities were mainly related to the teacher leading students to summarize the class content. The other format was for student representatives to reflect and summarize the learning of the lesson. Both approaches made it difficult to truly understand the overall knowledge acquisition. ⑤ In the Evaluation-Reflection stage, teachers had to analyze students' online learning and offline classroom performance separately, and both the workload and difficulty of analyzing the data were high. Based on the relationship of teacher-student interaction, a teacher-student interaction mechanism was proposed in the first round of research, guided by generative learning theory. This interaction mechanism is shown in Fig. 5.

### Second round in design-based research: interaction behaviors and supporting tools

**Fig. 6:**
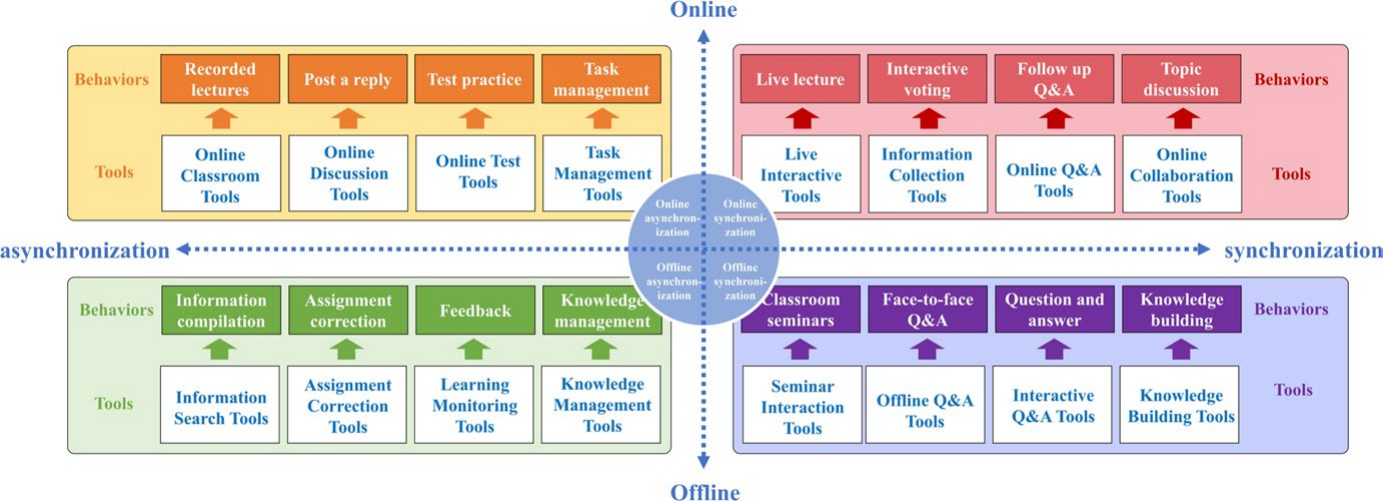
Teacher-student interaction behavior and tool support based on online learning spaces

The teaching content of the second round was Online Teaching and Learning During Pandemic Prevention and Content Analysis. The design and implementation of student–teacher interactions followed the model of student–teacher interactions in online learning spaces. Based on the above problems of teacher-student interactions in online learning spaces, typical teacher-student interaction behaviors and supporting tools in different interaction forms were analyzed and summarized, as shown in Fig. 6.

Teachers and students used tools to carry out activities in the environment, resulting in different behaviors. The specific representations of 16 teacher-student interaction behaviors are depicted in Fig. 6 and it was examined how teachers and students address each other in each teacher-student interaction behavior. Representative interaction tools were provided for the characteristics and needs of each teacher-student interaction behavior, to improve the quality of this interaction. The details of the specific tools and their functions are listed in Table 2.

**Table 2:** The functions of specific tools

Tools	Functions
Online classroom tools	Online classroom tools offer a large number of high-quality training courses that can create a classroom that supports timely interactions between teachers and students
Online discussion tools	Online discussion tools can support online issues, discussions, comments, and replies. Favorite views can be praised and shared
Online test tools	Online test tools are convenient for teachers to release tasks and remind students to carry out group activities while helping teachers understand the situation and progress of tasks carried out by each group
Task management tools	Task management tools can prompt students to carry out team cooperation, help students to build a knowledge framework, and stably achieve curriculum goals by establishing periodic projects
Live interactive tools	Live interactive tools can create classes for live broadcasts, which is convenient for online management. At the same time, during the live broadcast, teachers and students can interact by raising their hands and asking questions
Information collection tools	Information collection tools integrate online questionnaires, online tests, and data analysis, thus helping teachers and students carry out questionnaire surveys, examination evaluations, voting, elections, and other activities
Online Q&A tools	Online Q&A tools support the posting of replies to questions and support gamified teaching and learning assessments
Online collaboration tools	Online collaboration tools can support multiple students online and edit documents in time
Information search tools	Information search tools can provide readers with searching, online reading, and downloading of Chinese academic literature and foreign language literature
Assignment correction tools	Assignment correction tools can intelligently correct English compositions online, thus supporting interactive and automatic correction of assignments
Learning monitoring tools	Learning monitoring tools can provide both teaching and learning processes with data-based and intelligent information support
Knowledge management tools	Knowledge management tools can prompt students to carry out team cooperation, help students to build a knowledge framework, and stably achieve curriculum goals by establishing periodic projects
Seminar interaction tools	Seminar interaction tools can support the creation of workshops and promote student–teacher interaction
Offline Q&A tools	Offline Q&A tools can support students to scan a code and input their own questions and comments, making the conversation direct and deep
Interactive Q&A tools	Interactive Q&A tools can support interactive Q&A between teachers and students in offline classes
Knowledge building tools	Knowledge building tools offer a variety of templates, such as mind maps and flowcharts, to support knowledge management

### Third round in design-based research: model implementation strategy

The teaching content of the third round was Statistical Processing of Research Data and Educational Technology Action Research. Based on the model of teacher-student interaction in online learning spaces, the teacher-student interaction was designed and implemented. To address the problems of teacher-student interactions identified in the first two rounds of the design-based study, the following implementation strategies were adopted.

#### Personalized independent study

The feature of supporting personalized learning in online learning spaces was adopted to design the pre-learning stage. This stage allowed students to identify and express existing problems or propose new problems from contexts or experiences. The teacher used the course platform to guide students through individualized self-learning in the form of question guides and topic responses before class, thus encouraging students to identify, ask, and solve problems.

#### Collaborative thinking visualization

Using the online learning spaces to perceive contexts and enrich resources, teachers guided students to externalize both the state and quality of collaborative knowledge construction. This, in turn, helped teachers to understand how students communicate, collaborate, and negotiate in collaborative knowledge construction activities.

#### Multi-platform data connectivity

Teachers connected the MOOC platform of China University and the MU Classroom platform to collect data on students' online learning on the MOOC platform of China University and offline classroom interaction on the MU Classroom platform. Teachers also comprehensively analyzed students' online and offline learning, to dynamically adjust the teaching content and teaching activities.

## Results

### Interaction S-T curve and the result of Flanders interaction analysis

The effects of the teacher-student interaction model were examined using the real-time dynamic AI analysis system for S-T curve of teacher-student interaction analysis and Flanders interaction analysis.

*Interaction S-T curve.* The teacher-student interaction curve provides a visual representation of teacher-student interaction, where the line along the horizontal axis represents the speaking teacher and the line along the vertical axis represents speaking students. When the curve is skewed towards the horizontal axis, teacher activity is pre-leading; when it is skewed towards the vertical axis, student activity is pre-leading. A 45-degree curve indicates sufficient teacher-student interaction during this period (Yuan & Ou, 2011). The S-T analysis method is mainly used to quantitatively analyze and evaluate the teaching and learning process. In this study, the ratio of student to teacher speech duration was defined as the quality of the teacher-student interaction. A lesson was partitioned into 20 segments with 2 min per segment, and three variables were constructed to describe student–teacher interactions, as shown in Table 3.

**Table 3:** Comparison of teacher-student interaction curves in a typical lesson

Variables	Formula	Definitions
Percentage of student speak time	$\mathop \sum \limits_{i = 1}^{20} Row\;(i) \div Total$	Ratio of the length of student speech in a lesson, including discussions and questions
Percentage of teacher speak time	$\mathop \sum \limits_{j = 1}^{20} Row\;(j) \div Total$	Ratio of the teacher's speaking time in a lesson, including questions and instructions
Quality of student–teacher interaction	$\mathop \sum \limits_{i = 1}^{20} Row\;(i) \div \mathop \sum \limits_{j = 1}^{20} Row\;(j)$	Ratio of student–teacher speaking time in a class

In this study, the videos of teaching activities of the following two online courses were sampled and analyzed: Overview of Educational Technology Research Methods and Educational Technology Action Research. Table 4 shows a comparison of the teacher-student interaction curves of the two classes.

**Table 4:**
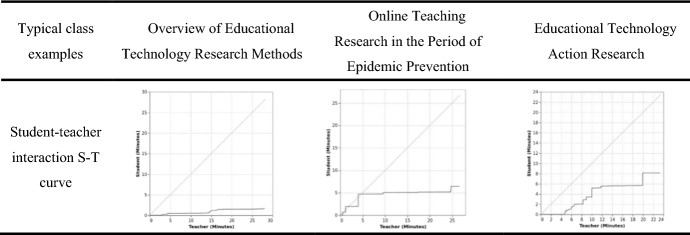
Comparison of teacher-student interaction curves in typical classes

Table 3 shows typical examples of each round of design-based research, which are briefly summarized as follows: ① in the first round of design-based research, the typical lesson Overview of Educational Technology Research Methods, the teacher-student interaction curve tended to be horizontal. This shows that the teacher dominated the class, while students spoke less and simply responded to the teacher's questions; therefore, the teacher-student interaction was lacking. The calculated quality of student–teacher interactions was 0.10. ② In the second round of design-based research, the typical lesson Online Teaching Research in the Period of Pandemic Prevention, the teacher-student interaction curve could be fitted to a 45° line within the first 5 min. This shows that there was a brief interaction between the teacher and students at the beginning, but the teacher still dominated the classroom most of the time. While the students spoke more than in the first round, this was still not enough. The calculated quality of student–teacher interactions was 0.24. ③ In the third round of design-based research, the typical lesson Educational Technology Action Research, the teacher-student interaction curves fitted the 45° line steadily from 4 to 12 min, showing vertical and horizontal replacement in the rest of the time. This indicates that the desired teacher-student interaction was achieved. The calculated quality of student–teacher interactions was 0.36.

In summary, the results of the comparison of the S-T curves of teacher-student interactions in three typical class cases showed that the teacher-student interaction model in online learning spaces can effectively improve teacher-student interaction and enhance the quality of teacher-student interactions.

*Flanders interaction analysis* After three rounds of design-based research, the real-time dynamic AI analysis system was used to automatically collect various teaching data generated in the classroom. A report of this analysis is shown in Table 5.

**Table 5:** Flanders teacher-student interaction analysis data

Indicators	1st round	2nd round	Reference	Definitions
Percentage of teacher talk (TT)	0.89	0.72	**0.68**	Proportion of the time the teacher talks to the total instructional time
Percentage of pupil talk (PT)	0.09	0.26	**0.20**	Proportion of the time pupils talk to the total instructional time
Teacher response rate (TRR)	0.42	0.62	**0.42**	Ratio of the teacher's feedback of the student's time to the teacher's non-teaching time
Stable state ratio (SSR)	0.79	0.82	**0.50**	Ratio of discourse time in which teacher-student talk remains for more than 3 s to the total instructional time
Pupil stable state ratio (PSSR)	0.04	0.21	**0.35**	Percentage of student's discourse time that lasts for more than 3 s as a percentage of the student's discourse time

Bold values are from the real-time dynamic AI analysis system

The data showed that: ① the steady state ratio (SSR) values were similar in both rounds of the design-based study, indicating that the teacher-student interaction remained relatively stable. ② Compared with the first round, the second round specified the content and form of teacher-student interaction. Additionally, the percentage of teacher talk (TT) decreased, while the percentage of pupil talk (PT) and pupil steady state ratio (PSSR) increased. This result indicates that the teacher paid more attention to the students' learning experience and returned the initiative to the students. ③ The teacher response rate (TRR) increased, indicating that teachers coordinated the cooperative learning status of each group and were able to make targeted suggestions more efficiently.

In summary, the results of the Flanders interaction analysis showed that, after three iterations of refinement, the TT value decreased, PT and PSSR increased, and TRR improved. This indicates that the teacher-student interaction model in online learning spaces could effectively improve teacher-student interactions and enhance the quality of teacher-student interactions.

### Pre-test and post-test of the innovative problem-solving ability result

**Fig. 7:**
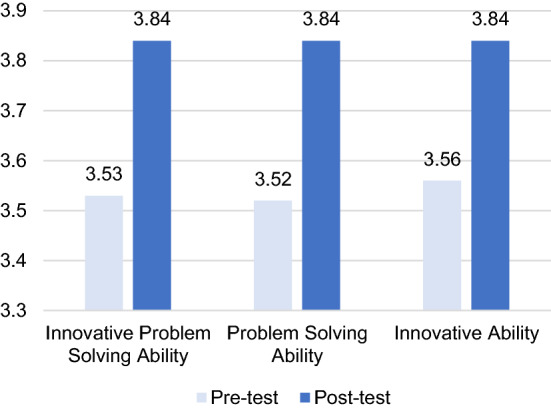
Histogram of the mean scores of the overall pre-test and post-test of innovative problem-solving ability

Pre-tests and post-tests on students’ innovative problem-solving ability were compared before and after the three-iteration design-based study using paired samples t-tests conducted via SPSS 26.0 (see Table 6). A histogram of the mean scores of the overall pre-test and post-test of innovative problem-solving ability is shown in Fig. 7.

**Table 6:** Paired-sample t-test results for the pre-test and post-test of innovative problem-solving ability

Pre-test and post-test	Paired differential					t	df	p
	Mean	SD	Std. Error Mean	95% confidence interval of the difference				
				Lower limit	Higher limit			
Innovative problem-solving ability	− .31	.62	.11	− .54	− .08	− 2.7	30	.010*
Problem-solving ability	− .32	.65	.12	− .56	− .08	− 2.7	30	.011*
Innovation ability	− .28	.66	.12	− .53	− .04	− 2.4	30	.023*

^*^The significance threshold is 0.05

**Fig. 8:**
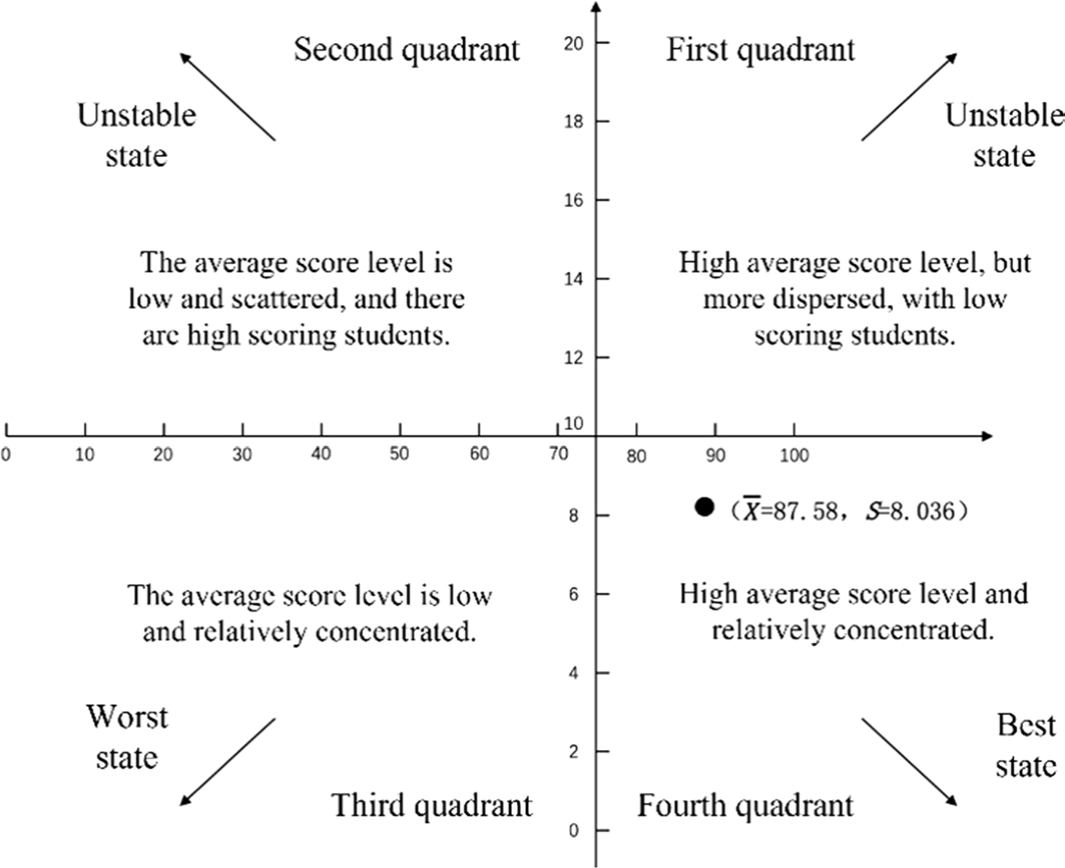
‾*X*-*S* plane analysis model

As shown in Table 6 and Fig. 8, the mean value of innovative problem-solving ability of experimental subjects was 3.53 in the pre-test and 3.84 in the post-test. The innovative problem-solving ability of experimental subjects was significantly improved after the application of the model (*t* (30) = – 2.74, *p* = 0.010). The mean score of problem-solving ability in the pre-test was 3.53 and the mean score in the post-test was 3.84. The problem-solving ability of experimental subjects was significantly improved after the application of the model (*t* (30) = – 2.71, *p* = 0.011). The mean score of innovation ability of the experimental subjects was 3.56 in the pre-test and 3.84 in the post-test. The innovation ability of the experimental subjects was also significantly improved after the application of the model (*t* (30) = – 2.39, *p* = 0.023).

In conclusion, the teacher-student interaction model in the online learning spaces significantly improved students' innovative problem-solving ability.

### Overall characteristics of final examination results

The average score of the final exam reflects the concentration trend of the overall class level distribution, and the standard deviation reflects its degree of dispersion. By forming a two-dimensional plane with the mean and standard deviation of the final exam, the overall characteristics of the grades can be comprehensively and visually presented. The descriptive statistics of the course final exam results are shown in Table 7.

**Table 7:** Descriptive statistics of final exam results

	N	Minimum value	Maximum value	Average value	Standard deviation
Final Exam Results	31	67	96	87.58	8.036
Number of Active Cases	31				

The ${\bar{\rm X}}$-S plane analysis model was utilized to comprehensively and visually examine the overall characteristics of the experimental subjects’ course final exam scores. In this model, the horizontal coordinates indicate the magnitude of the mean score, the vertical coordinates indicate the magnitude of the standard deviation, and the origin of the coordinates take the norm (*X* = 75, *S* = 10) as reference standard. The two parameters ${\bar{X}}$ and *S* are used to determine in which quadrant of the plane the achievement samples fall into. Different quadrants represent groups of achievement samples with different statistical characteristics.

As shown in Fig. 8, the mean and standard deviation divided the overall characteristics of samples into four quadrants. The samples in the first and second quadrants were more dispersed, while the samples in the third and fourth quadrants were more concentrated. The sample mean in the first and fourth quadrants was higher, while in the second and third quadrants, the mean was lower. If the mean and standard deviation coordinates of the class final exam scores are located in the fourth quadrant, the scores of the final exam are high on average (Xie & Li, 2017).

The mean score of the course final exam in this study is *X* = 87.58 and the standard deviation is *S* = 8.036; (*X* = 87.58, *S* = 8.036) is located in the fourth quadrant of the ${\bar{\rm X}}$-S plane analysis model. Combined with the histogram of course final exam scores, this indicates that the course final exam scores of the sampled subjects were high on average.

## Discussion

Through theoretical and practical research, a set of theoretical frameworks of teacher-student interaction was established based on online learning spaces. Further, the principles of teacher-student interaction and the functional support of online learning spaces for teacher-student interaction were clarified, and a teacher-student interaction model in the online learning spaces was constructed. Moreover, the interaction mechanism, behavior and tool support, and implementation strategies about the model were further improved through design-based research. In addition, the effectiveness of the model was verified, teacher-student interaction was improved, the activity of teacher-student interaction was increased, and students' innovative problem-solving skills were enhanced.

Song et al. (2020) developed a teacher-student interaction measurement model in online teaching from the two perspectives of pedagogical existence and social existence. Sybing (2021) constructed a conversational model for teacher-student interaction, which could determine the dialogue opportunities arising during in-class interactions as well as the relevant characteristics of active dialogue development. Compared with these existing studies, the present study achieved two major advances. First, based on theoretical analysis and current research, the teacher-student interaction model in the online learning spaces was constructed. A specific focus was directed on interaction mechanism, behaviors and tool support, as well as implementation strategies. Second, AI and big data techniques were used to analyze the activity of teacher-student interaction in practice. Based on the real-time dynamic AI analysis system, the analysis of student–student interaction could be visualized, which greatly improved the scientific and timeliness of the analysis of student–student interactions.

Certain aspects of this study still need to be improved. For example, in teaching practice, different teaching contents may impose different requirements on teacher-student interaction; therefore, the effective interaction between teachers and students could be explored according to the requirements of different teaching contents to further improve the integrity of the study. Effect evaluation showed that the ability to reflect on problems and the ability to communicate and express could not be greatly improved within a short period of time. Thus, the improvement effect between before and after the application of the model was not significant. Consequently, further research is needed that addresses how the model can be improved continuously. Considering these limitations, a follow-up study will develop the theoretical research and practical application further based on the construction of the teacher-student interaction model in online learning spaces.

## Conclusion

The engaged interaction between teachers and students is central for promoting teaching quality. In this study, literature research and design-based research were adopted to build and test a teacher-student interaction model in online learning spaces. First, a preliminary model was developed by analyzing the principles behind the interaction between teachers and students and the support features of online learning spaces. Additionally, the interaction model was refined during three rounds of teaching practices. The results of three teacher-student interaction analyses showed that the model significantly promoted students' engagement during the interaction and was also able to significantly improve students' final exam scores and innovation problem-solving ability. This model provides new ideas and methods for the integration and innovative application of online learning spaces in the information era.
